# Supplementary material for: HIVprotI: an integrated web based platform for prediction and design of HIV proteins inhibitors
Source: J Cheminform. 2018 Mar 9;10:12. doi: 10.1186/s13321-018-0266-y (PMC5845081; doi:10.1186/s13321-018-0266-y)
Supplement: Supplementary file 2 — Additional file 2. Source code of HIVProtI web server. [file 13321_2018_266_MOESM2_ESM.zip › HIVprotI_Source-code/hivprotI/weka-3-6-6/doc/weka/core/neighboursearch/kdtrees/package-summary.html]

weka.core.neighboursearch.kdtrees


---


|  |  |  |
| --- | --- | --- |
| **Overview** \| **Package** \| Class \| **Tree** \| **Deprecated** \| **Index** \| **Help** \| **Weka's home** | |  |
| **PREV PACKAGE**   **NEXT PACKAGE** | **FRAMES**    **NO FRAMES**     **All Classes** |  |


---

## Package weka.core.neighboursearch.kdtrees

| **Class Summary** | |
| --- | --- |
| **KDTreeNode** | A class representing a KDTree node. |
| **KDTreeNodeSplitter** | Class that splits up a KDTreeNode. |
| **KMeansInpiredMethod** | The class that splits a node into two such that the overall sum of squared distances of points to their centres on both sides of the (axis-parallel) splitting plane is minimum.    For more information see also:    Ashraf Masood Kibriya (2007). |
| **MedianOfWidestDimension** | The class that splits a KDTree node based on the median value of a dimension in which the node's points have the widest spread.    For more information see also:    Jerome H. |
| **MidPointOfWidestDimension** | The class that splits a KDTree node based on the midpoint value of a dimension in which the node's points have the widest spread.    For more information see also:    Andrew Moore (1991). |
| **SlidingMidPointOfWidestSide** | The class that splits a node into two based on the midpoint value of the dimension in which the node's rectangle is widest. |

---


|  |  |  |
| --- | --- | --- |
| **Overview** \| **Package** \| Class \| **Tree** \| **Deprecated** \| **Index** \| **Help** \| **Weka's home** | |  |
| **PREV PACKAGE**   **NEXT PACKAGE** | **FRAMES**    **NO FRAMES**     **All Classes** |  |


---
